# TRIM24-RIP3 axis perturbation accelerates osteoarthritis pathogenesis

**DOI:** 10.1136/annrheumdis-2020-217904

**Published:** 2020-09-07

**Authors:** Jimin Jeon, Hyun-Jin Noh, Hyemi Lee, Han-Hee Park, Yu-Jin Ha, Seok Hee Park, Haeseung Lee, Seok-Jung Kim, Ho Chul Kang, Seong-il Eyun, Siyoung Yang, You-Sun Kim

**Affiliations:** 1 Department of Pharmacology, Ajou University School of Medicine, Suwon, Gyeonggi-do, Republic of Korea; 2 Department of Biomedical Sciences, Graduate School, Ajou University School of Medicine, Suwon, Republic of Korea; 3 CIRNO, Sungkyunkwan University, Suwon, Republic of Korea; 4 Department of Biochemistry & Molecular Biology, Ajou University School of Medicine, Suwon, Republic of Korea; 5 Department of Biological Sciences, Sungkyunkwan University, Suwon, Republic of Korea; 6 Intellectual Information Team, Future Medicine Division, Korea Institute of Oriental Medicine, Daejeon, Republic of Korea; 7 Department of Orthopaedic Surgery, Uijeongbu St. Mary’s Hospital, The Catholic University of Korea College of Medicine, Uijeongbu, Republic of Korea; 8 Department of Physiology, Ajou University School of Medicine, Suwon, Republic of Korea; 9 Department of Life Science, Chung-Ang University, Seoul, Republic of Korea

**Keywords:** osteoarthritis, therapeutics, arthritis

## Abstract

**Objectives:**

Recently, necroptosis has attracted increasing attention in arthritis research; however, it remains unclear whether its regulation is involved in osteoarthritis (OA) pathogenesis. Since receptor-interacting protein kinase-3 (RIP3) plays a pivotal role in necroptosis and its dysregulation is involved in various pathological processes, we investigated the role of the RIP3 axis in OA pathogenesis.

**Methods:**

Experimental OA was induced in wild-type or *Rip3* knockout mice by surgery to destabilise the medial meniscus (DMM) or the intra-articular injection of adenovirus carrying a target gene (Ad-Rip3 and Ad-Trim24 shRNA). RIP3 expression was examined in OA cartilage from human patients; Trim24, a negative regulator of RIP3, was identified by microarray and in silico analysis. Connectivity map (CMap) and in silico binding approaches were used to identify RIP3 inhibitors and to examine their direct regulation of RIP3 activation in OA pathogenesis.

**Results:**

RIP3 expression was markedly higher in damaged cartilage from patients with OA than in undamaged cartilage. In the mouse model, adenoviral RIP3 overexpression accelerated cartilage disruption, whereas *Rip3* depletion reduced DMM-induced OA pathogenesis. Additionally, TRIM24 knockdown upregulated RIP3 expression; its downregulation promoted OA pathogenesis in knee joint tissues. The CMap approach and in silico binding assay identified AZ-628 as a potent RIP3 inhibitor and demonstrated that it abolished RIP3-mediated OA pathogenesis by inhibiting RIP3 kinase activity.

**Conclusions:**

TRIM24-RIP3 axis perturbation promotes OA chronicity by activating RIP3 kinase, suggesting that the therapeutic manipulation of this pathway could provide new avenues for treating OA.

**Key messages**

**What is already known about this subject?**

- Osteoarthritis (OA) is a degenerative disease characterised by the destruction of articular cartilage.
- Necroptosis has attracted increasing attention; however, it is unclear whether its regulation is involved in OA pathogenesis.
- Receptor-interacting protein kinase-3 (RIP3) plays a pivotal role in necroptosis, and its dysregulation is involved in various pathological processes; however, its physiological and pathological roles in cartilage have not yet been addressed.

**What does this study add?**

- The TRIM24-RIP3 axis regulates RIP3-mediated OA pathogenesis in mice by modulating the expression of catabolic factors.
- The Connectivity map approach identified AZ-628 as a potent RIP3 inhibitor whose inhibition of RIP3 kinase activity abolished OA pathogenesis.

**How might this impact on clinical practice or future developments?**

- Modulation of the TRIM24-RIP3 axis could be a novel molecular mechanism of OA pathogenesis.
- RIP3 kinase inhibitors could be a potential clinical intervention for treating OA.

## Introduction

Osteoarthritis (OA), a degenerative disease characterised by articular cartilage destruction, is caused by anabolic and catabolic factor imbalance due to mechanical stress.^1^ These factors alter biochemical pathways in chondrocytes and reduce their ability to produce extracellular matrix (ECM), degrade ECM molecules via catabolic matrix-degrading enzymes and exert inflammatory responses.^2 3^ Increasing evidence suggests that chondrocyte death is involved in OA pathogenesis.^4 5^ Apoptosis is thought to eliminate dedifferentiated chondrocytes without releasing type II collagen or other ECM components.^6^ Necroptosis is a recently described form of pathophysiological cell death that causes the cell membrane to burst, exerting detrimental effects on surrounding tissues. Necroptotic cells release damage-associated molecular patterns that trigger robust inflammatory responses; thus, necroptosis is markedly more immunogenic than apoptosis and promotes inflammation and ECM degradation.^7^

Necroptosis is mainly mediated by receptor-interacting protein kinase 1 (RIP1), receptor-interacting protein kinase-3 (RIP3) and mixed lineage kinase domain-like protein (MLKL).^8 9^ Assembly and activation of the RIP1-RIP3 complex is dependent on the kinase activity of both proteins.^10–12^ RIP3 activation leads to the phosphorylation of MLKL, which translocates to and disrupts the cell membrane.^13–16^ RIP3’s complex role in cell death, inflammation, tumorigenesis and metabolism has been widely studied, as have tissue injury mediators and circulating markers of disease progression and severity.^17–19^ RIP1 and RIP3 inhibition reportedly improves the outcomes of numerous pathological mouse models, including kidney, brain and heart ischaemic–reperfusion injury; pancreatitis; and acetaminophen-induced hepatitis models.^8 20 21^ Moreover, RIP3 can act both dependently and independently of its substrate, MLKL, suggesting that RIP3 and MLKL may exert tissue-specific effects.^22^

A recent report suggested that RIP3 promotes arthritis pathogenesis via the TLR-TRIF-RIP3-IL-1β axis independent of MLKL^23^; another study found that the p55TNFR-IKK2-RIP3 axis orchestrates synovial fibroblast arthritogenic and cell death responses.^24^ However, the physiological and pathological roles of RIP3 in cartilage have not yet been addressed,^20 25^ and it remains unclear whether RIP3-mediated necroptosis regulation is involved in OA pathogenesis. Herein, we investigated the possible functions and underlying molecular mechanisms of RIP3 in OA pathogenesis using human OA cartilage samples and destabilisation of the medial meniscus (DMM) surgery-induced OA mouse models. We determined whether RIP3 promotes OA progression by activating necroptosis signalling in RIP3 knockout (KO) mice and used a connectivity map (CMap) approach to identify novel RIP3 inhibitors. We believe that our study paves the way for future research investigating RIP3 blockade as an optimal therapeutic strategy for OA.

## Methods

### Human OA cartilage samples and experimental OA mouse models

Human cartilage samples were obtained from individuals 63–80 years old undergoing total knee arthroplasty (online supplementary table S1). All patients provided written informed consent; sample collection was approved by the Institutional Review Board (IRB) of the Catholic University (UC14CNSI0150). Male C57BL/6 and Rip3^-/-^ mice (C57BL/6; provided by Dr V.M. Dixit, Genentech, San Francisco, USA) were housed in the Laboratory Animal Research Center of Ajou University and maintained according to the guidelines of its Institutional Animal Care and Use Committee, which approved all animal procedures. To produce experimental OA models, 12-week-old male mice were subjected to surgical DMM and sacrificed 10 weeks post-surgery; female mice were not used because of the effects of female hormones on OA pathogenesis.^26 27^ The following adenoviruses for intra-articular injection were purchased from Vector Biolabs (Malvern, USA): Ad-C (1060), Ad-Rip3 (ADV-270614) and Ad-shRNA Trim24 (shADV-274975). Wild-type (WT) mice were injected in the knee joint twice weekly with adenovirus (1×10^9^ PFUs/10 µL) and sacrificed 3 weeks after the first adenoviral injection.

10.1136/annrheumdis-2020-217904.supp1 Supplementary data

### Reagents

Antibodies were purchased from Enzo Biochem (New York, USA; RIP3), BD-Transduction Laboratories (Breda, Netherlands; RIP1), Santa Cruz Biotechnology (Dallas, USA; glyceraldehyde-3-phosphate dehydrogenase (GAPDH), Actin, inhibitor of nuclear factor kappa B (IκBα), Vimentin), Cell Signalling Technology (Danvers, USA; p-ERK, phospho-the c-jun n-terminal kinase (p-JNK), poly (ADP-ribose) polymerase (PARP), Caspase 3, phospho-Receptor-interacting protein kinase 1 (p-RIPK1), NIK), Abcam (Cambridge, UK; p-MLKL, p-RIP3), MLKL, TRIM24, cyclooxygenase-2 (COX2), matrix metalloproteinase 3 (MMP3), matrix metalloproteinase 13 (MMP13)) and Sigma-Aldrich (St Louis, USA; LC3B, Vinculin). Tumour necrosis factor-α (TNF-α) and zVAD were obtained from R&D Systems (Minneapolis, USA). Cycloheximide, Pepstatin A and MG132 were purchased from Calbiochem (San Diego, USA). Chloroquine diphosphate (CQ) and AZ-628 were obtained from Sigma-Aldrich. GSK’872 was purchased from Selleckchem (Houston, USA). Selumetinib was obtained from Abcam. Neratinib was purchased from MedChemExpress (Princeton, USA). HS-1371 was made in-house.^28^

### Statistical analysis

All experiments were independently performed at least four times. Two independent groups were compared using the Shapiro-Wilk normality test, Levene’s homogeneity of variance test and a two-tailed independent t-test. Multiple comparisons were made using the Shapiro-Wilk test, Levene’s test and one-way analysis of variance with Bonferroni’s post hoc test. Data based on ordinal grading systems were analysed using non-parametric Mann-Whitney U tests.

## Results

### RIP3 overexpression-mediated gene signatures are associated with OA

**Figure 1:**
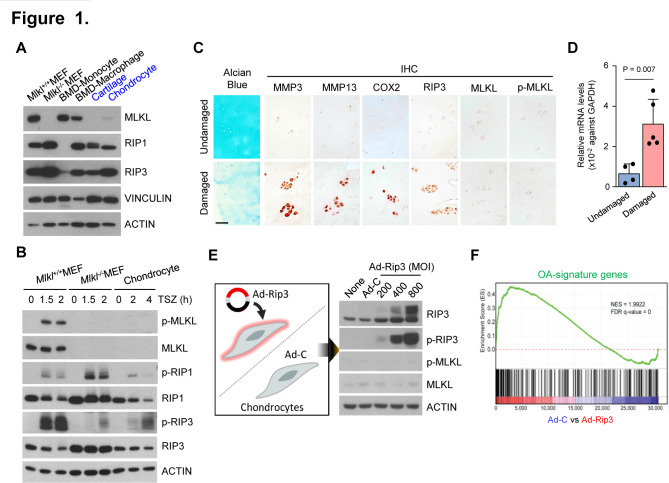
Non-canonical role of receptor-interacting protein kinase-3 (RIP3) in chondrocytes is related to osteoarthritis (OA) pathogenesis. (A) Expression levels of necroptosis regulators in chondrocytes. Proteins extracted from wild-type and mixed lineage kinase domain-like protein (MLKL) knockout mouse embryonic fibroblasts (MEFs), bone marrow-derived monocytes (BMDMs), macrophages, cartilage tissue and primary mouse articular chondrocytes were analysed by western blotting (*n*=3). (B) MEFs and chondrocytes were treated with TSZ (TNF+zVAD+SMAC mimetic) and cell lysates analysed by western blotting (*n*=3). (C) Undamaged and damaged human cartilage stained with Alcian blue, Safranin O, and RIP3, p-MLKL and MLKL immunostaining (*n*=5). Scale bar: 100 µm. (D) *RIP3* expression in undamaged and damaged human OA cartilage measured by quantitative PCR (*n*=5). Values are expressed as the mean±SEM. Statistical analyses were performed using two-tailed t-tests. (E) Western blot analysis of RIP3, p-RIP3, MLKL and p-MLKL in chondrocytes infected with Ad-C (control) or Ad-Rip3 at the indicated multiplicity of infection (MOI) (*n*=3). (F) Gene set enrichment analysis of RIP3-related genes (listed in online supplementary table S2) in treated cells compared with controls.

To investigate the role of necroptosis in cartilage degeneration, we examined RIP3 and MLKL expression patterns in various mouse tissue samples. Although RIP3 expression did not differ significantly between tissues, MLKL expression was extremely low in cartilage (online supplementary figure S1A, B) and primary mouse articular chondrocytes, which are the predominant cell type in cartilage (figure 1A; online supplementary figure S1C). Low MLKL expression was not due to protein insolubility, constitutive protein turnover or protein stability since neither proteasome (MG132) nor lysosome (CQ, E64d/Pep A) inhibitors changed the MLKL protein levels (online supplementary figure S1D, E). Prototypical necroptotic stimuli (TNF-α+zVAD+SMAC mimetic; TSZ) can activate RIP3, which phosphorylates MLKL to induce necroptosis.^15^ Following TSZ treatment, MLKL phosphorylation was detected strongly in mouse embryonic fibroblast (MEF) cells but not in chondrocytes (figure 1B; online supplementary figure S1F, G), whereas TNF failed to induce necroptosis in chondrocytes, likely due to defective MLKL phosphorylation (online supplementary figure S1H).

To explore the possible role of RIP3 in cartilage, we examined RIP3 expression in human OA undamaged and damaged cartilage samples. RIP3 expression was significantly higher in damaged OA cartilage than in undamaged samples (figure 1C, D; online supplementary figure S2A), whereas MLKL and p-MLKL expression did not differ and was undetectable. Next, we expressed RIP3 ectopically in primary mouse articular chondrocytes infected with Ad-Rip3. RIP3 overexpression did not cause MLKL phosphorylation (figure 1E; online supplementary figure S2B), which can induce necroptosis^29 30^; thus, RIP3 overexpression in OA pathogenesis may be distinct from its canonical necroptosis-dependent functions. Studies suggested that upregulated RIP3 expression leads to RIP1-independent necroptosis through MLKL phosphorylation.^29 31^ Nevertheless, to test this, we overexpressed RIP3 in RIP1-deficient MEFs to show that RIP3 activation is sufficient to trigger the downstream events. Upregulated RIP3 expression potentiated MLKL phosphorylation in the absence of RIP1 phosphorylation, suggesting that the role of RIP3 in OA development is unlikely necroptosis independent (online supplementary figure S2C).

Next, we performed genome-wide expression profiling using microarrays to investigate transcriptomic changes in Ad-Rip3-infected chondrocytes. RIP3 overexpression increased the expression of catabolic factors—matrix metalloproteinase 3 (MMP3; online supplementary figure S2D)—that play key roles in OA pathogenesis, inflammation, MMP activation and ECM degradation.^32^ Functional enrichment analysis revealed that RIP3 overexpression-induced genes involved in TNF-α signalling and inflammation (online supplementary figure S2E). To further assess RIP3-induced gene expression, we examined the expression of 150 upregulated and 71 downregulated genes in OA cartilage (online supplementary table S2)^33^ by Gene set enrichment analysis (GSEA), finding that OA-signature genes were upregulated in RIP3-overexpressing chondrocytes (figure 1F).

### RIP3 modulates OA pathogenesis

**Figure 2:**
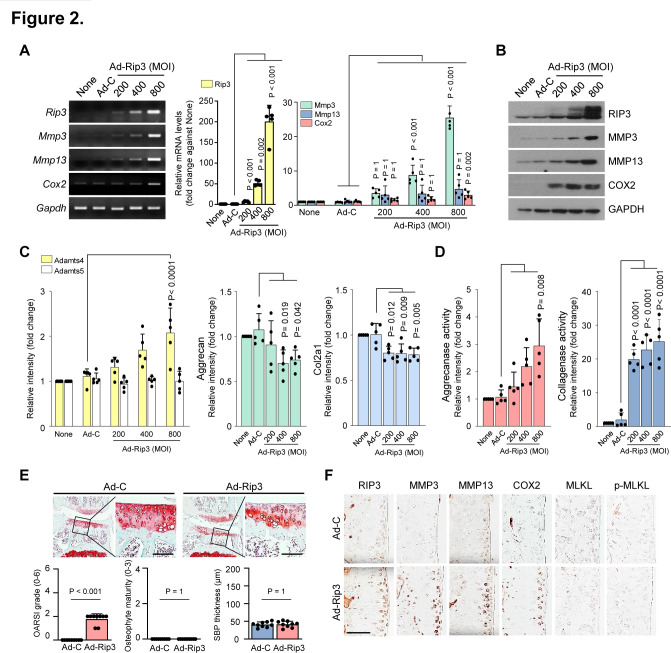
Modulation of receptor-interacting protein kinase-3 (RIP3) expression is correlated with osteoarthritis (OA). (A and B) Reverse transcription PCR (RT-PCR) (left), quantitative PCR (qPCR) (right) (A) and western blot (B) analysis of RIP3, matrix metalloproteinase 3 (MMP3), matrix metalloproteinase 13 (MMP13) and COX2 in chondrocytes infected with Ad-C or Ad-Rip3 at the indicated multiplicity of infection (MOI) (*n*=5). (C) qPCR analysis of *Adamts4, Adamts5, aggrecan* and *Col2a1* in Ad-Rip3-infected chondrocytes (*n*=5). (D) Aggrecanase (left) and collagenase (right) activity by Ad-Rip3 infection in chondrocytes (*n*=5). (E) Cartilage destruction and OA development in mouse knee joints intra-articularly injected with Ad-C and Ad-Rip3 assessed by Safranin-O staining, Osteoarthritis Research Society International (OARSI) grading, osteophyte formation and subchondral bone plate thickness (*n*=9). (F) RIP3, MMP3, MMP13, COX2, MLKL and p-MLKL immunostaining in Ad-C-injected or Ad-Rip3-injected cartilage. Statistical analyses were conducted using one-way analysis of variance with Bonferroni’s test (A, C and D), non-parametric Mann-Whitney U tests (E, OARSI grading, osteophyte formation) or two-tailed t-tests (E, subchondral bone plate thickness). Scale bar: 100 μm.

**Figure 3:**
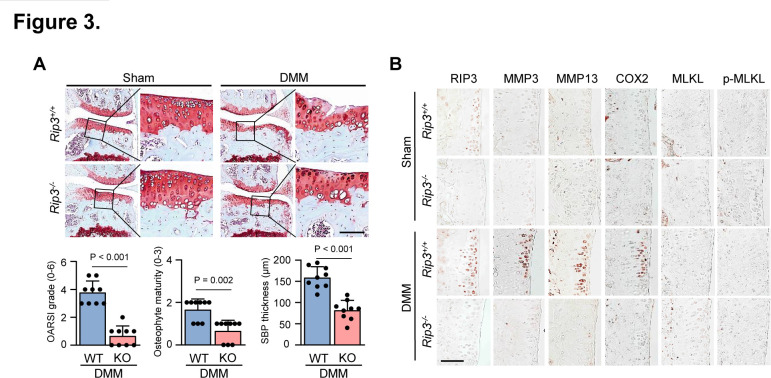
*Rip3* knockout (KO) mice displayed reduced osteoarthritis (OA) pathogenesis. (A and B) Wild-type (WT) and *Rip3* knockout mice were subjected to surgical destabilise the medial meniscus (DMM) (*n*=9). Cartilage destruction was assessed by Safranin-O staining, OARSI grading, osteophyte formation, subchondral bone plate thickness (A), and receptor-interacting protein kinase-3 (RIP3), matrix metalloproteinase 3 (MMP3), matrix metalloproteinase 13 (MMP13), COX2, mixed lineage kinase domain-like protein (MLKL) and p-MLKL immunohistochemical staining (B). Values are expressed as the mean±SEM of the indicated number of independent experiments. Statistical analyses were conducted using one-way analysis of variance with Bonferroni’s test (A), a non-parametric Mann-Whitney U test (A, OARSI grading, osteophyte formation) or a two-tailed t-test (A, subchondral bone plate thickness). Scale bar: 100 μm.

MMP3, MMP13, ADAMTS4 and ADAMTS5 are known to play a crucial role in OA pathogenesis; COX2 is primarily involved in inflammation and eventually leads to cartilage matrix degradation by collagenase and aggrecanase activation.^1 34^ We next investigated the association between elevated RIP3 expression and OA pathogenesis, finding upregulated catabolic factor (MMP3, MMP13, COX2 and ADAMTS4) and downregulated anabolic factor (Col2a1 and Aggrecan) expression in articular chondrocytes (figure 2A–C; online supplementary figure S3A, B), which are all known to disrupt OA cartilage.^35^ Moreover, MMP3 and MMP13 have collagenase activity; Adamts4 and Adamts5 primarily function as aggrecanase 1 and 2, respectively.^2 6^ We found that aggrecanase and collagenase activity is upregulated by Ad-Rip3 infection (figure 2D). Consistent with our findings in chondrocytes, Ad-Rip3 induced severe cartilage destruction and OA symptoms in WT mice compared with Ad-C (figure 2E), with immunohistochemical staining indicating that Ad-Rip3 triggered RIP3 overexpression in cartilage and increased MMP3, MMP13 and COX2 expression (figure 2F; online supplementary figure S3C). To verify these effects, we established DMM-induced OA models, most suitable for human OA development,^36^ in *Rip3*-deficient mice. Cartilage destruction, OA manifestations and catabolic factor expression were markedly reduced in these mice compared with WT mice (figure 3A, B; online supplementary figure S3D). Consistent with RIP3 upregulation in human OA cartilage, RIP3 deficiency was found to attenuate OA in the DMM-induced mouse model, suggesting that RIP3 plays a key role in OA pathogenesis.

Since OA and elevated RIP3 expression are both strongly associated with cell death,^29 30^ we investigated whether RIP3 overexpression triggers the cell death pathway to accelerate OA pathogenesis by examining cytotoxicity in Ad-Rip3-infected chondrocytes. Ad-Rip3-induced RIP3 overexpression did not alter chondrocyte morphology or lactate dehydrogenase (LDH) release (online supplementary figure S3E) or induce PARP cleavage, despite TNF-mediated apoptosis-induced PARP and caspase 3 cleavage (online supplementary figure S3F), suggesting that cell death is not implicated in RIP3-mediated OA pathogenesis. However, RIP3 overexpression did induce TNF-mediated signalling (online supplementary figure S3G), indicating that RIP3 upregulation may potentiate OA pathogenesis via non-canonical MLKL-independent functions.

### TRIM24 negatively regulates RIP3-mediated OA pathogenic signatures

**Figure 4:**
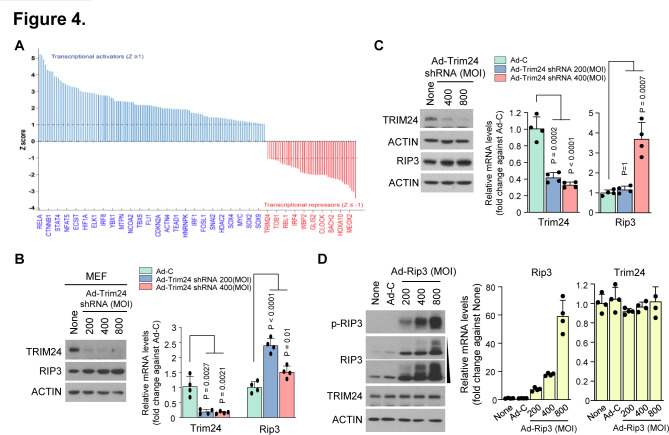
RIP3 overexpression alters gene expression patterns via the negative regulator TRIM24. (A) Transcriptional regulators were analysed by Ingenuity Pathway Analysis (IPA) software (QIAGEN, https://www.qiagenbioinformatics.com/products/ingenuitypathway-analysis) to predict upstream regulators based on microarray data. Analysed data were filtered by activation Z-score >1 or <-1, and regulators were not required to be expressed in the samples analysed. (B) Mouse embryonic fibroblasts (MEFs) and (C) chondrocytes were infected with Ad-C or Ad-Trim24 shRNA at the indicated multiplicity of infection (MOI). Cell lysates were analysed by western blotting (left) (*n*=3) and quantitative PCR (qPCR) (right) (n=4). (D) Western blot analysis of RIP3, p-RIP3 and TRIM24 in chondrocytes infected with Ad-C or Ad-Rip3 (left) (*n*=3). qPCR analysis of *Rip3* and *Trim24* in chondrocytes infected with Ad-C or Ad-Rip3 (right) (*n*=4). Values are expressed as the mean±SEM. Statistical analyses were performed using two-tailed t-tests.

To understand the mechanism underlying RIP3-mediated alterations in chondrocyte molecular patterns, we performed Ingenuity Pathway Analysis to predict the upstream regulators responsible for such changes. Consequently, we found 367 differentially expressed gene transcripts (figure 4A). Among the 69 negative upstream regulators, we selected TRIM24, an important transcriptional coregulator that modulates TNF-α signalling via Nuclear factor kappa B (NF-κB)^37^ and interacts with retinoic acid receptor alpha (RARα) in a ligand-dependent manner to attenuate RARα-mediated transcription in mice.^38^ Although previous studies report the function of TRIM24 in other tissues and diseases,^39–41^ TRIM24 modulation is still undefined in cartilage and other joint tissues. To determine whether TRIM24 is a negative upstream regulator of RIP3 transcription, we knocked down its expression in primary mouse articular chondrocytes using Ad-Trim24 shRNA. Although TRIM24 downregulation enhanced *Rip3* mRNA expression (figure 4B, C; online supplementary figure S4A, B), RIP3 overexpression did not affect TRIM24 mRNA or protein expression (figure 4D; online supplementary figure S4C), indicating that TRIM24 is a negative upstream regulator of RIP3 transcription.

**Figure 5:**
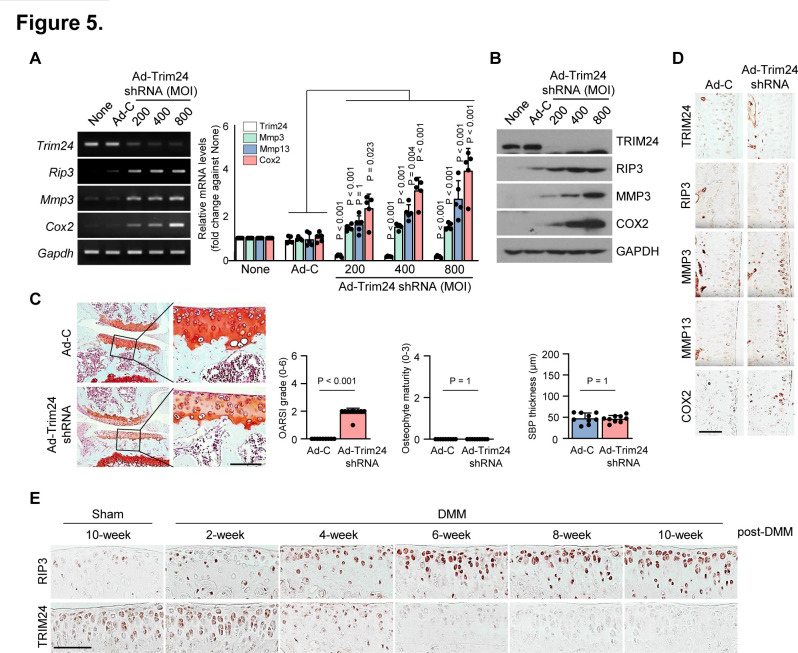
TRIM24 is an upstream regulator of receptor-interacting protein kinase-3 (RIP3) expression. (A and B) Reverse transcription PCR (RT-PCR) (left), quantitative PCR (qPCR) (right) and western blot (B) analysis of TRIM24, RIP3, matrix metalloproteinase 3 (MMP3) and COX2 in chondrocytes infected with Ad-C or Ad-Trim24 shRNA at the indicated multuplicity of infection (MOI) (*n*=5). (C and D) Knee joint cartilage of wild-type (WT) mice intra-articularly injected with Ad-C or Ad-Trim24 shRNA stained with Safranin-O (*n*=9). (C) Cartilage destruction, osteophyte formation and subchondral bone thickness were assessed by scoring. (D) TRIM24, RIP3, MMP3, matrix metalloproteinase 13 (MMP13) and COX2 immunostaining in cartilage injected with Ad-C or Ad-Trim24 shRNA. (E) Relative expression levels of the indicated proteins were determined from immunohistochemistry of cartilage every 2 weeks after destabilise the medial meniscus (DMM) surgery (*n*=9). Values are expressed as the mean and were analysed by one-way analysis of variance with Bonferroni’s test (A), a non-parametric Mann-Whitney U test (C; Osteoarthritis Research Society International (OARSI) grading, osteophyte formation), or a two-tailed t-test (C; subchondral bone plate thickness). Scale bar: 100 μm.

Next, we investigated whether regulating RIP3 transcription via TRIM24 knockdown in MEFs elicits OA-related gene expression patterns. Ad-Trim24 shRNA-infected MEFs displayed increased *Mmp3*, *Mmp13* and *Cox2* mRNA expression, as well as RIP3 and COX2 protein expression (online supplementary figure S4D). Similar to this, Ad-Trim24 shRNA-infected chondrocytes displayed also increased RIP3, MMP3, MMP13 and COX2 expression (figure 5A, B; online supplementary figure S4E, F), suggesting that RIP3 could be a molecular target of TRIM24-mediated negative regulation. Ad-Trim24 shRNA-mediated TRIM24 knockdown in the knee joint tissues of mice markedly increased cartilage destruction, osteophyte formation, subchondral bone plate thickness (figure 5C) and catabolic factor expression (MMP3, MMP13 and COX2; figure 5D; online supplementary figure S5A). To clarify the contribution of the TRIM24-RIP3 axis to OA disease onset or progression, we examined the time-course expression profiles of Trim24 and RIP3 in mouse OA cartilage samples after DMM surgery. OA manifestations, such as cartilage destruction, osteophyte formation and subchondral bone sclerosis, were gradually increased at 4~6 weeks after DMM surgery (online supplementary figure S5B), whereas Trim24 was decreased in the early stages of OA pathogenesis and RIP3 expression was increased at OA onset (figure 5E; online supplementary figure S5C). Thus, TRIM24 may negatively regulate elevated RIP3 expression to accelerate OA pathogenesis.

### Identification of drugs that correlate with RIP3 expression using CMap

**Figure 6:**
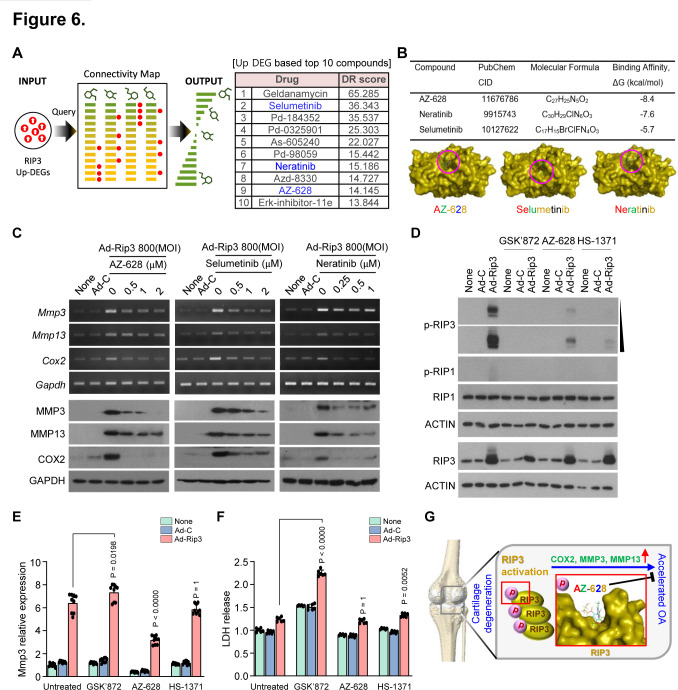
AZ-628 attenuated receptor-interacting protein kinase-3 (RIP3) kinase activity and reduced RIP3-mediated osteoarthritis (OA) pathogenesis. (A) Schematic illustration of in silico drug screening using a Connectivity map (CMap) approach and the top 10 predicted compounds. (B) Binding affinities of AZ-628, neratinib and selumetinib to RIP3 using AutoDock Vina. (C) Matrix metalloproteinase 3 (MMP3), matrix metalloproteinase 13 (MMP13) and COX2 expression determined by reverse transcription PCR (upper) and western blotting (lower) in chondrocytes infected with Ad-C or Ad-Rip3 in the presence/absence of AZ-628, selumetinib or neratinib at the indicated dose (*n*=5). (D) Inhibition of RIP3 kinase activity by AZ-628. Chondrocytes were infected with adenovirus Ad-C or Ad-Rip3 and treated with GSK’872, AZ-628 and HS-1371. Cell lysates were analysed by western blotting (*n*=3). (E) Quantitative PCR analysis of *Mmp3* under the same conditions as (C) (*n*=9). (F) Cell cytotoxicity assessed using an lactate dehydrogenase (LDH) release assay (*n*=6). (G) Diagram of RIP3-mediated OA and the inhibitory effects of AZ-628. Values are expressed as the mean±SEM. Statistical analyses were performed using two-tailed t-tests.

Targeting TRIM24 is risky in drug development because of the diverse functions of TRIM24 as a transcriptional coregulator.^40 42^ Therefore, we identified drugs to block OA pathogenesis by inhibiting RIP3 using in silico compound screening with a CMap approach, as described previously.^43 44^ Briefly, we searched the drug-induced transcriptome data of ~20 000 small molecules from the CMap database for compounds with opposite expression signatures following RIP3 overexpression, prioritising downregulated genes that were upregulated by RIP3 overexpression in chondrocytes (figure 6A). We identified the following candidate compounds that have not been previously associated with OA: selumetinib, neratinib and AZ-628 (figure 6A; online supplementary figure S6A). Molecular docking analysis revealed that nine compounds had good RIP3 binding affinity (binding energy <−7.0 kcal/mol) and five had moderate RIP3 interactions (binding energy >−7.0 kcal/mol; figure 6B).

We tested the cytotoxic effect of these compounds in chondrocytes and found that AZ-628 and selumetinib had no toxicity at 2 µM, whereas neratinib had no toxicity at 1 µM (online supplementary figure S6B). Next, we examined whether these compounds inhibited RIP3-mediated OA pathogenesis, finding that they reduced RIP3 overexpression-induced MMP3, MMP13 and COX2 expression in a dose-dependent manner (figure 6C; online supplementary figure S6C, D), suggesting that these compounds may inhibit RIP3 activity. Since AZ-628 displayed higher binding affinity than selumetinib or neratinib and inhibited the OA manifestations in RIP3-overexpressing chondrocytes more effectively (figure 6C), it was investigated further. High RIP3 expression leads to its spontaneous autophosphorylation and may potentiate OA pathogenesis; thus, OA could be attenuated by inhibiting RIP3 kinase activity with anticancer drugs—dabrafenib (DAB) and sorafenib—targeting RIP3 kinase.^20^

### RIP3 kinase inhibition abrogates Rip3 activity in OA pathogenesis

Known RIP3 kinase inhibitors have a type I (DAB and GSK’843), II (sorafenib, GSK’067 and HS-1371), III, or unclassified (GSK’872, GSK’840) kinase binding mode,^20^ whereas DAB and HS-1371 have shown potential in the treatment of RIP3-mediated inflammatory diseases.^28^ To examine their ability to regulate RIP3, we investigated the in vitro binding affinity of DAB, GSK’872, HS-1371 and AZ-628 (online supplementary figure S7A, B) and examined their effects on RIP3-overexpressing chondrocytes. All four compounds almost completely inhibited RIP3 phosphorylation (figure 6D; online supplementary figure S7C), yet *Mmp3* and *Cox2* expression only decreased in AZ-628-treated chondrocytes (figure 6E; online supplementary figure S7D). GSK’872 and HS-1371 exerted greater cytotoxic effects on the chondrocytes than AZ-628 (figure 6F; online supplementary figure S7E), suggesting that their binding may have caused conformational changes in RIP3 and induced chondrocyte apoptosis (online supplementary figure S7E). Collectively, our data suggest that AZ-628 is a potent RIP3 kinase inhibitor that could block RIP3-mediated OA pathogenesis. TRIM24-RIP3 axis perturbation accelerated OA pathogenesis by altering gene expression but not RIP3-mediated necroptotic cell death in chondrocytes, while RIP3 kinase activity inhibition by AZ-628 attenuated OA-related gene expression without chondrocyte cytotoxicity. Thus, the involvement of RIP3 kinase activity in cartilage pathophysiology suggests that modulating RIP3 expression and activity may help treat OA (figure 6G; online supplementary figure S7F).

## Discussion

RIP3 is a crucial regulator of necroptosis with roles in various human diseases—inflammatory bowel disease, multiple sclerosis and toxic epidermal necrosis (TEN).^29^ Previously, we demonstrated that elevated RIP3 expression in keratinocytes from patients with TEN leads to spontaneous autophosphorylation and inopportune necroptosis via MLKL phosphorylation.^29 30^ Lee *at al* reported that interferon-γ (IFN-γ)-deficient collagen-induced arthritic mice showed exacerbation of cartilage damage and joint inflammation as well as acceleration of MLKL, RIP1 and RIP3 production in the joints.^45^ Recent reports suggested a possible link between cartilage injury and necroptotic processes, depending on oxidative stress and cytokine release in OA disease.^46 47^ These studies immunohistochemically analysed the expression of necroptosis markers—RIP3, MLKL and p-MLKL—as evidence of the presence of necroptotic chondrocytes in fractured human and murine cartilage. However, most studies have focused on manipulating the RIP3-MLKL axis to regulate necroptosis and disease processes; however, our findings suggest that RIP3 plays an MLKL-independent role in cartilage. Moreover, in TRIM24-knockdown knee joints, TRIM24 was found to negatively regulate RIP3 transcription, with TRIM24 loss upregulating RIP3-mediated changes in molecular patterns of OA pathogenesis. Although TRIM24 negatively regulated RIP3 expression, further studies are required to elucidate how/why TRIM24 is downregulated during OA. Nevertheless, RIP3 activation remains a key factor for promoting OA pathogenesis alongside catabolic factor expression.

Since upregulated RIP3 expression is related to disease pathology via either MLKL-dependent necroptotic cell death or altered gene expression under both MLKL-dependent and independent conditions, it is important to regulate RIP3 activity in RIP3 hyperactivation-driven diseases.^30 48^ Known RIP3 kinase inhibitors have type I, II or III kinase binding modes; however, few RIP3 inhibitors have been discovered and the development of novel RIP3 kinase inhibitors has been limited as highly flexible and unpredictable inhibitor-induced changes in RIP3 conformation may increase apoptosis.^49^ A recent study proposed that tyrosine kinase inhibitors could be repurposed to develop RIP3 kinase inhibitors with off-target effects in a low micromolar-nanomolar range.^50^ Despite difficulties separating the primary on-target and secondary off-target effects against RIP3 kinase activity, drug repurposing could allow the development of novel drugs with faster clinical application.

Several RIP1 inhibitors are currently in clinical trials for rheumatoid arthritis,^51^ suggesting that off-target or specific RIP3 kinase inhibitors could be used to treat OA. The drug AZ-628, which was found to correlate with RIP3 expression, is a novel pan-Raf inhibitor whose cross-reactivity profile suggests that it may have similar functions to DAB.^52^ Cellular arrays suggested that AZ-628 affects RIP3 kinase activity to prevent RIP3 phosphorylation without chondrocyte cytotoxicity and reduces the molecular pattern of OA pathogenesis. Thus, our study is the first to demonstrate that AZ-628 inhibits RIP3 activation and reduces OA pathogenesis without chondrocyte cytotoxicity. Although GSK’872 and HS-1371 are potent RIP3 kinase inhibitors, they exerted cytotoxic effects in chondrocytes, which may complicate their clinical development. Thus, the therapeutic potential of RIP3 inhibitors in OA should be considered further. Despite many specificity and safety factors that must be overcome before kinase inhibitors can be repurposed to treat OA, RIP3 blockade remains a promising therapeutic strategy for OA.
