# Supplementary material for: TRIM24-RIP3 axis perturbation accelerates osteoarthritis pathogenesis
Source: Ann Rheum Dis. 2020 Sep 7;79(12):1635–43. doi: 10.1136/annrheumdis-2020-217904 (PMC7677493; doi:10.1136/annrheumdis-2020-217904)
Supplement: Supplementary data [file annrheumdis-2020-217904supp001.pdf]

SUPPLEMENTARY DATA

TRIM24-RIP3 axis perturbation accelerates osteoarthritis pathogenesis

Jimin Jeon[1,2,3†], Hyun-Jin Noh[2,4†], Hyemi Lee[1,2,3], Han-Hee Park[2,4], Yu-Jin Ha[2,4], Seok Hee Park[3,5], Haeseung Lee[6], Seok-Jung Kim[7], Ho Chul Kang[2,8], Seong-il Eyun[9], Siyoung Yang[1,2,3*], and You-Sun Kim[2,4*]

[1]Department of Pharmacology, Ajou University School of Medicine, Suwon, 16499, Korea; [2]Department of Biomedical Sciences, Graduate School, Ajou University School of Medicine, Suwon, 16499, Korea; [3]CIRNO, Sungkyunkwan University, Suwon, 16419, Korea; [4]Department of Biochemistry & Molecular Biology, Ajou University School of Medicine, Suwon, 16499, Korea; [5]Department of Biological Sciences, Sungkyunkwan University, Suwon, 16419, Republic of Korea; [6]Intellectual Information Team, Future Medicine Division, Korea Institute of Oriental Medicine, Daejeon 34054, Korea; [7]Department of Orthopaedic Surgery, Uijeongbu St. Mary's Hospital, The Catholic University of Korea College of Medicine, Uijeongbu, 11765, Korea; [8]Department of Physiology, Ajou University School of Medicine, Suwon, 16499, Korea; [9]Department of Life Science, Chung-Ang University, Seoul, 06974, Korea

[†] These authors contributed equally to this work.

*Correspondence to:

Siyoung Yang, Ph.D.

Department of Pharmacology, Ajou University, School of Medicine,
164 Worldcup-ro, Yeongtong-gu, Suwon, 16499, Korea
Tel.: 82-31-219-5065; Fax: 82-31-219-5069; E-mail: yangsy@ajou.ac.kr

AND

You-Sun Kim

Department of Biochemistry & Molecular Biology, Ajou University, School of Medicine

164 Worldcup-ro, Yeongtong-gu, Suwon, 16499, Korea
Tel.: 82-31-219-4509; Fax: 82-31-219-4530; E-mail: yousunkim@ajou.ac.kr

Supplementary Materials and Methods

Supplementary Tables S1–S3

Supplementary Figures S1–S7

Supplementary References

SUPPLEMENTARY MATERIALS AND METHODS

Primary mouse articular chondrocytes and cell culture

Mouse articular chondrocytes were isolated from the cartilage of postnatal day 5 ICR mice, enzymatically digested with proteinase and collagenase, as described previously,[1] and maintained in DMEM (Capricorn scientific GmbH; Hessen, Germany) supplemented with 10 % FBS, 100 units/mL of penicillin, and 100 μg/mL of streptomycin. As described previously,[2] on day 3, the cells (4.25×10^5 cells/well) were infected with adenovirus or treated with recombinant proteins, as indicated. *Mlkl*$^{+/+}$ and *Mlkl*$^{-/-}$ MEFs were maintained in DMEM supplemented with 10 % FBS and penicillin-streptomycin.

Cell viability assay

Cell viability was assessed using a lactate dehydrogenase (LDH) colorimetric assay kit (BioVision (Milpitas, CA, USA)). Chondrocytes were seeded into a 96-well dish (1.5×10^4 cells/well), incubated for 24 h (5% CO2, 37 °C), and treated with various concentrations of AZ-628, selumetinib, and neratinib for 24 h before the supernatant was analyzed using a microplate reader at 495 nm. Untreated (100 % viability) and Triton X-100 treated (0 % viability) samples were used for normalization. Percentage viability was calculated as follows:

$$100 - \frac{sample\,LDH\, - \,negative\ control}{\max LDH\, - \,negative\ control} \times 100.$$

Western blotting

Cells were lysed in M2 buffer,[3] and mouse tissues were lysed in a lysis buffer composed of 50 mM Tris-HCl (pH 7.5), 150 mM NaCl, 50 mM NaF, 1 % Tween 20, 0.2 % NP-40, and

protease inhibitors. Equal amounts of cell extracts were resolved by SDS-PAGE (6% stacking gels and 10% running gels) and analyzed by immunoblotting.

Reverse transcription (RT)-PCR and qPCR

Total RNA was extracted from articular chondrocytes using TRIzol reagent (Molecular Research Center (Cincinnati, OH, USA)), reverse transcribed into complementary DNA (cDNA) using ImProm-II™ Reverse Transcriptase (Promega (Madison, WI, USA)), and amplified by PCR or qPCR with primers as summarized in Supplementary Table S3. qPCR was performed using SYBR premix Ex Taq (TaKaRa Bio, Kusatsu, Shiga, Japan), with results normalized to Gapdh and expressed as fold-changes relative to the control.

Collagenase and aggrecanase activity assay

Chondrocytes were seeded in 6-well dishes (2×10^5 cells·well) and infected with Ad-C or Ad-Rip3. The cells were incubated for 36 h in DMEM without fetal bovine serum (FBS). The culture medium was collected; equal volumes were concentrated using Viva® Spin Columns (Sartorius Stedim Biotech, Göttingen, Germany) according to the manufacturer's protocol. Concentrated samples were assayed for total collagenase activity using EnzCheck™ Gelatinase/Collagenase Assay kits (Molecular Probes, Eugene, OR, USA). Collagenase activity was measured as fluorescent signals using the SYNERGY H1 microplate reader (BioTek Instruments, Inc., Winooski, VT, USA) at Ex/Em = 485/530 nm. Aggrecanase activity was assessed using an aggrecanase activity assay kit (Abnova, Taipei, Taiwan). The aggrecanase level was quantified in concentrated supernatant by measuring the absorbance at 430 nm according to the manufacturer's protocol.

Histology and immunohistochemistry

Human OA cartilage and mouse knee joints were fixed in 4 % paraformaldehyde and embedded in paraffin. Mouse knee joints were decalcified for 2 weeks in 0.5 M EDTA (pH 7.4). Paraffin-embedded samples were stained with Safranin-O or Alcian blue or immunostained. Cartilage destruction was assessed in the experimental OA mouse model by three observers blinded to the experimental groupings and scored according to the OARSI (Osteoarthritis Research Society International) grading system (grade 0–6). OARSI scores were presented as the mean maximum score for each mouse. Representative Safranin-O staining images were selected from the most advanced lesions in each section and osteophyte maturity quantified as described previously.[4] Subchondral bone sclerosis was determined by measuring subchondral bone plate thickness. Immunohistochemical staining was performed in human and mouse cartilage sections with MMP3, MMP13, and MLKL (Abcam), COX2 and TRIM24 (Proteintech), and RIP3 (Enzo Biochem) antibodies.

Microarray analyses

Mouse articular chondrocytes were infected with Ad-Rip3 or Ad-C (MOI, 800) for 36 h. Total RNA was isolated using TRIzol reagent (Molecular Research Center) and analyzed with an Affymetrix Mouse GeneChip 2.0 ST Array (Macrogen, Seoul, Korea) according to the manufacturer's protocol. Microarray data have been deposited in the Gene Expression Omnibus under accession codes GSE154669 (http://www.ncbi.nlm.nih.gov/geo/query/acc.cgi?acc=GSE154669) (for Rip3).

***In silico* binding assay**

The chemical structures of the ligands (Supplementary Fig. 5A) used were retrieved from the PubChem database (https://pubchem.ncbi.nlm.nih.gov).[5] Molecular docking analyses were performed using AutoDock Vina (ver. 1.1.2)[6] which is widely used to determine protein-

ligand binding affinities and positions. All docking analyses were conducted with rigid receptors and fully flexible ligands. Receptor coordinates and docking parameters were prepared using AutoDock MGLTools (ver. 1.5.6).[7] Ligand binding affinities were evaluated using the negative Gibbs free energy (ΔG; kcal/mol). Docking structures were visualized using PyMOL (ver. 1.3; DeLanoScientific, San Carlos, CA, USA).

Gene set enrichment analysis (GSEA)

GSEA was performed using java GSEA software (ver. 4.0.3; Broad Institute, MIT).[8] Genes were ranked according to their expression; those up- or downregulated following RIP3 overexpression were selected as the RIP3-related gene set.

Statistical analysis

All experiments were performed independently at least four times. Two independent groups were compared using the Shapiro-Wilk normality test, Levene's homogeneity of variance test, and a two-tailed independent t-test. Multiple comparisons were made using the Shapiro-Wilk test, Levene's test, and one-way analysis of variance with Bonferroni's post-hoc test. Data based on ordinal grading systems were analyzed using non-parametric Mann-Whitney U tests. P values < 0.05 were considered statistically significant.

SUPPLEMENTARY TABLES

Table S1. Characteristics of specimens from patients with OA

No.	Age/gender	ICRS[a] grade	Joint	Weight (kg)	Height (m)	BMI[b] (kg/m^2)	Use
1	65/F	4	Knee	158	53	21.23	IHC/qPCR
2	80/F	4	Knee	143.3	55.1	26.83	IHC/qPCR
3	72/F	4	Knee	165	65	23.88	IHC/qPCR
4	63/F	4	Knee	152	52	22.51	IHC/qPCR
5	69/F	4	Knee	151	60	26.31	IHC/qPCR
6	73/F	4	Knee	153.8	70.75	29.89	IHC
7	63/F	4	Knee	156	72.2	29.67	IHC
8	73/F	4	Knee	154	83	35	IHC
9	75/F	4	Knee	154	55	23.19	IHC
10	63/F	4	Knee	163	74.3	27.96	IHC

[a]ICRS, International Cartilage Repair Society; [b]BMI, Body Mass Index

Table S2 Osteoarthritis signature genes[9]

Genes upregulated in osteoarthritic cartilage (150 genes)						
3830406C13Rik	Cdkn2b	Ebf3	Hey2	Mtss1	Rab23	Tgfbi
Abracl	Cdkn3	Egr2	Hhipl1	Ncapg	Rcan1	Tmem100
Adamts14	Cenpf	Epha3	Hmga2	Nedd4l	Rhbdl2	Tmem119
Adamts5	Cenpk	Eva1a	Homer2	Nedd9	S100a4	Tmem200a
Adamts6	Cep55	Evi2a	Hunk	Ngf	Sema3c	Tmem59l
Adgrg1	Chst13	Fam132b	Ier3	Nt5e	Serpine1	Tnfaip6
Adtrp	Cited4	Fam167a	Iqgap3	Ntf3	Serpine2	Tnfrsf12a
AI661453	Ckb	Fam60a	Itga3	Ociad2	Sgk1	Tom1l1
Akr1c20	Clic3	Fat3	Kcne4	Ogn	Sik1	Top2a
Anln	Col13a1	Fgf9	Kcnn4	Osbpl3	Slc2a5	Trim36
Arhgap44	Col18a1	Fhl2	Kcns3	P3h2	Slc38a5	Uroc1
Arl4a	Col1a1	Foxf1	Kif20a	Pamr1	Slc6a6	Vcan
Arntl2	Col7a1	Fstl3	Lamb3	Pcdh10	Slitrk6	Veph1
Aspm	Cpeb2	Fzd10	Lif	Pcdh18	Sntb1	Vwc2
Aspn	Csdc2	Galnt7	Lmo2	Pgm2l1	Sqrdl	Wisp1
Atrnl1	D330045A20Rik	Gja1	Lrrc8c	Plaur	St6galnac5	Wnt5a
B3gnt2	Diras1	Gjb2	Lrrc8e	Plekhg1	Stx1a	Zfp365
B3gnt5	Dkk3	Glis3	Lum	Popdc3	Syt11	Zfp367
Bmpr1b	Dnajc12	Glrb	Map1b	Postn	Sytl2	
C1galt1	Dner	Gmnn	Mob3b	Prex2	Tbx3	
Car12	Dsg2	Gpc4	Moxd1	Ptges	Tenm3	
Cdk1	Dusp4	Gria2	Msx2	R3hdml	Tfpi	

Genes downregulated in osteoarthritic cartilage (71 genes)						
Agtr2	Cmtm5	Evx1	Il17rb	Pde3b	Sgsm1	Tmem176b
Alx4	Cmya5	Fam198a	Il18bp	Piezo2	Slc14a1	Tnfrsf4
Apol9b	Col11a2	Fbln7	Kif1a	Ppp1r1b	Slc25a27	Wnk2
Atp1b2	Col16a1	Fgf14	Lgi4	Prx	Slitrk4	Zcchc5
C530008M17Rik	Crim1	Frzb	Lrrtm2	Ptger3	Sncg	Zfp385c
Cacna1c	Cyp39a1	Gpc5	Mpped2	Rarres2	Srl	
Cacna2d2	Dact1	Gprc5b	Myh14	Rcan2	Steap4	
Capn6	Dcc	Grin2c	Myoz3	Rflna	Stk32b	
Cdhr1	Ddit4	Gucy1a3	Nfam1	Rspo3	Tac1	
Ces1a	Erich3	Hmgcll1	Nrxn2	Sdc3	Tceal5	
Chrdl2	Esr1	Igf2	Obscn	Sez6l	Tmem176a	

Table S3. Primer sequences for qPCR and RT-PCR

Gene	Origin	Strand	Sequence	Size (bp)	AT[a] (°C)
ACAN	Mouse	[b]S<br>[c]As	5'-GAAGACGACATCACCATCCAG-3'<br>5'-CTGTCTTTGTCACCCACACATG -3'	581	55
Adamts4	Mouse	S<br>As	5'-CATCCGAAACCCTGTCAACTTG -3'<br>5'-GCCCATCATCTTCCACAATAGC -3'	281	58
Adamts5	Mouse	S<br>As	5'-ATGTCGTGCGTCAAGTTATGG-3'<br>5'-TCAGTCCCATCCGTAACCTTTG -3'	292	58
Col2a1	Mouse	S<br>As	5'-CACACTGGTAAGTGGGGCAAGA-3'<br>5'-GGATTGTGTTGTTTCAGGGTTCG-3'	173	55
Cox2	Mouse	S<br>As	5'-GGTCTGGTGCCTGGTCTGATGAT-3'<br>5'-GTCCTTTCAAGGAGAATGGTGC-3'	724	63
Gapdh	Mouse	S<br>As	5'-TCACTGCCACCCAGAAGAC-3'<br>5'-TGTAGGCCATGAGGTCCAC-3'	450	60
Mmp3	Mouse	S<br>As	5'-CTGTGTGTGGTTGTGTGCTCATCCTAC-3'<br>5'-GGCAAATCCGGTGTATAATTCACAATC-3'	350	58
Mmp13	Mouse	S<br>As	5'-TGATGGACCTTCTGGTCTTCTGGC-3'<br>5'-CATCCACATGGTTGGGAAGTTCTG-3'	473	58
Rip3	Human	[b]S<br>[c]As	5'- ATGTCGTGCGTCAAGTTATGG-3'<br>5'- CATAGGAAGTGGGGCTACGAT-3'	138	60
Rip3	Mouse	S<br>As	5'-CAGTGGGACTTCGTGTCCG-3'<br>5'-CAAGCTGTGTAGGTAGCACATC-3'	157	60
Trim24	Mouse	S<br>As	5'-CGAATGAAACTCATGCAACAACA-3'<br>5'-AGGTGCCGTAACCTGTATGTAA-3'	152	60

[a]AT, annealing temperature; [b]S, sense primer; [c]As, antisense primer

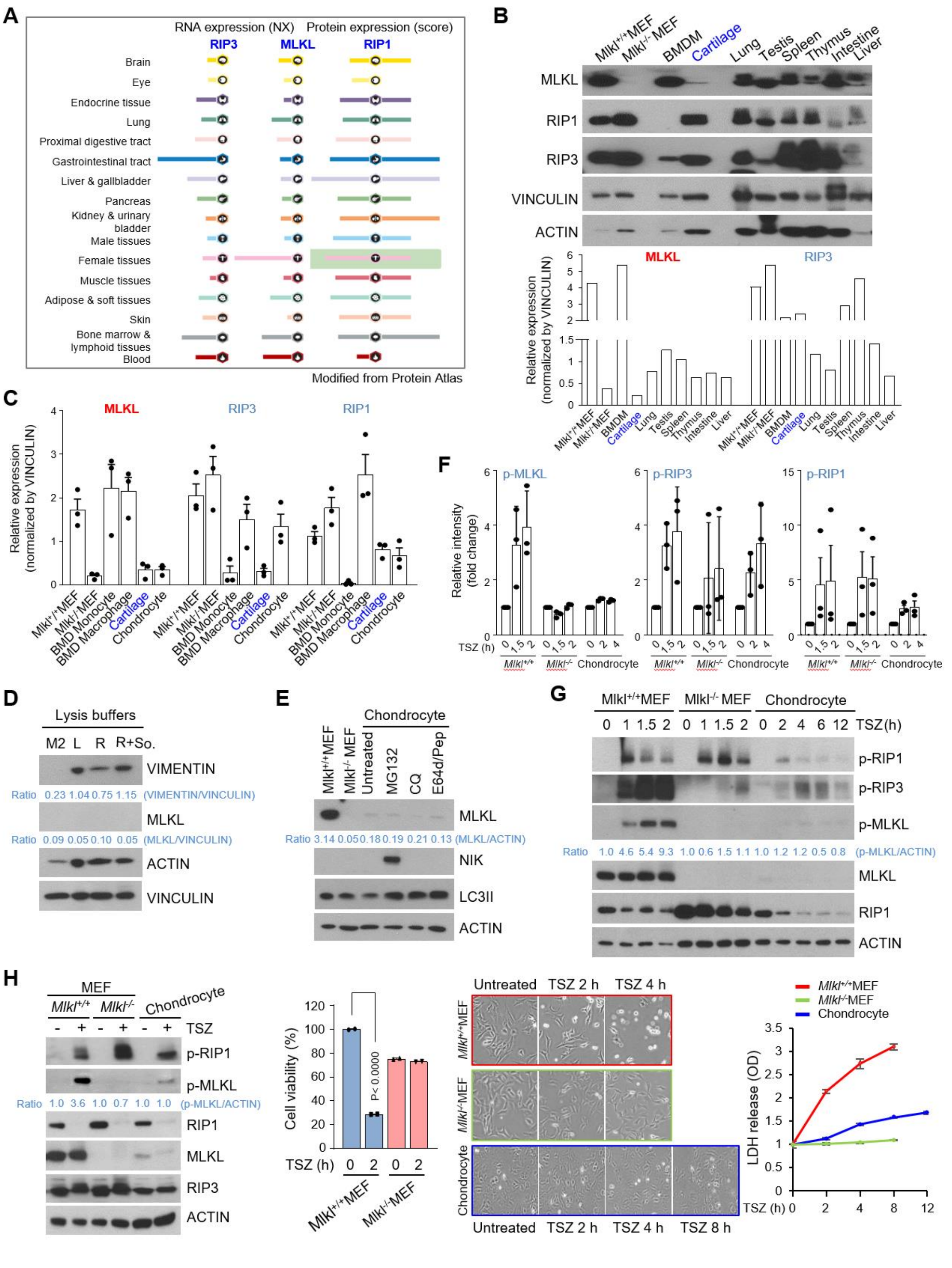
A
RNA expression (NX) Protein expression (score)
RIP3
MLKL
RIP1
Brain
Eye
Endocrine tissue
Lung
Proximal digestive tract
Gastrointestinal tract
Liver & gallbladder
Pancreas
Kidney & urinary bladder
Male tissues
Female tissues
Muscle tissues
Adipose & soft tissues
Skin
Bone marrow & lymphoid tissues
Blood
Modified from Protein Atlas
B
MLKL
RIP1
RIP3
VINCULIN
ACTIN
Relative expression (normalized by VINCULIN)
C
MLKL
RIP3
RIP1
D
Lysis buffers
M2 L R R+So.
VIMENTIN
MLKL
ACTIN
VINCULIN
E
Chondrocyte
Untreated
MG132
CQ
E64d/Pep
NIK
LC3II
F
p-MLKL
p-RIP3
p-RIP1
Relative intensity (fold change)
TSZ (h)
Chondrocyte
G
Chondrocyte
TSZ(h)
H
MEF
TSZ
Cell viability (%)
Untreated
TSZ 2 h
TSZ 4 h
TSZ 8 h
LDH release (OD)

Figure S1. Undetectable MLKL expression in chondrocytes. (**A**) Protein Atlas data showing tissue RIP1, RIP3, and MLKL expression patterns (modified from Protein Atlas). (**B**) Expression levels of necroptosis regulators in various tissue samples. Protein extracts were analyzed by western blotting (upper panel). Quantification of western blotting (bottom panel). (**C**) Quantification of western blotting results in figure 1A (*n*=3). (**D**) Chondrocytes were lysed in M2 buffer, Laemmli SDS buffer (L), RIPA buffer (R), or RIPA buffer with sonication (R+So.). Lysates were analyzed by western blotting. Vimentin was the positive control for insoluble protein detection (*n*=3). (**E**) Chondrocytes were treated with MG132, CQ, or 10 mg/mL of E64d plus 10 mg/mL Pepstatin A for 6 h, and cell lysates were analyzed by western blotting. NIK and LC3II were controls for blocking proteasome- and lysosome-dependent degradation, respectively (*n*=3). **(F)** Quantification of western blotting results in figure 1B (*n*=3). **(G)** MEFs and chondrocytes were treated with TSZ (TNF + zVAD + SMAC mimetic) for the indicated times, and cell lysates were analyzed by western blotting (*n*=3). **(H)** MEFs and chondrocytes were treated with TSZ (TNF + zVAD + SMAC mimetic), and cell lysates were analyzed by western blotting. (left panel) (*n*=3). MEFs and chondrocytes were treated with TSZ for the indicated times and cytotoxicity analyzed by MTT assays or phase-contrast microscopy (middle). TNF-induced cell death was also analyzed by LDH assays (right). Values are expressed as the mean ± SEM. Statistical analyses were performed using a two-tailed *t*-test.

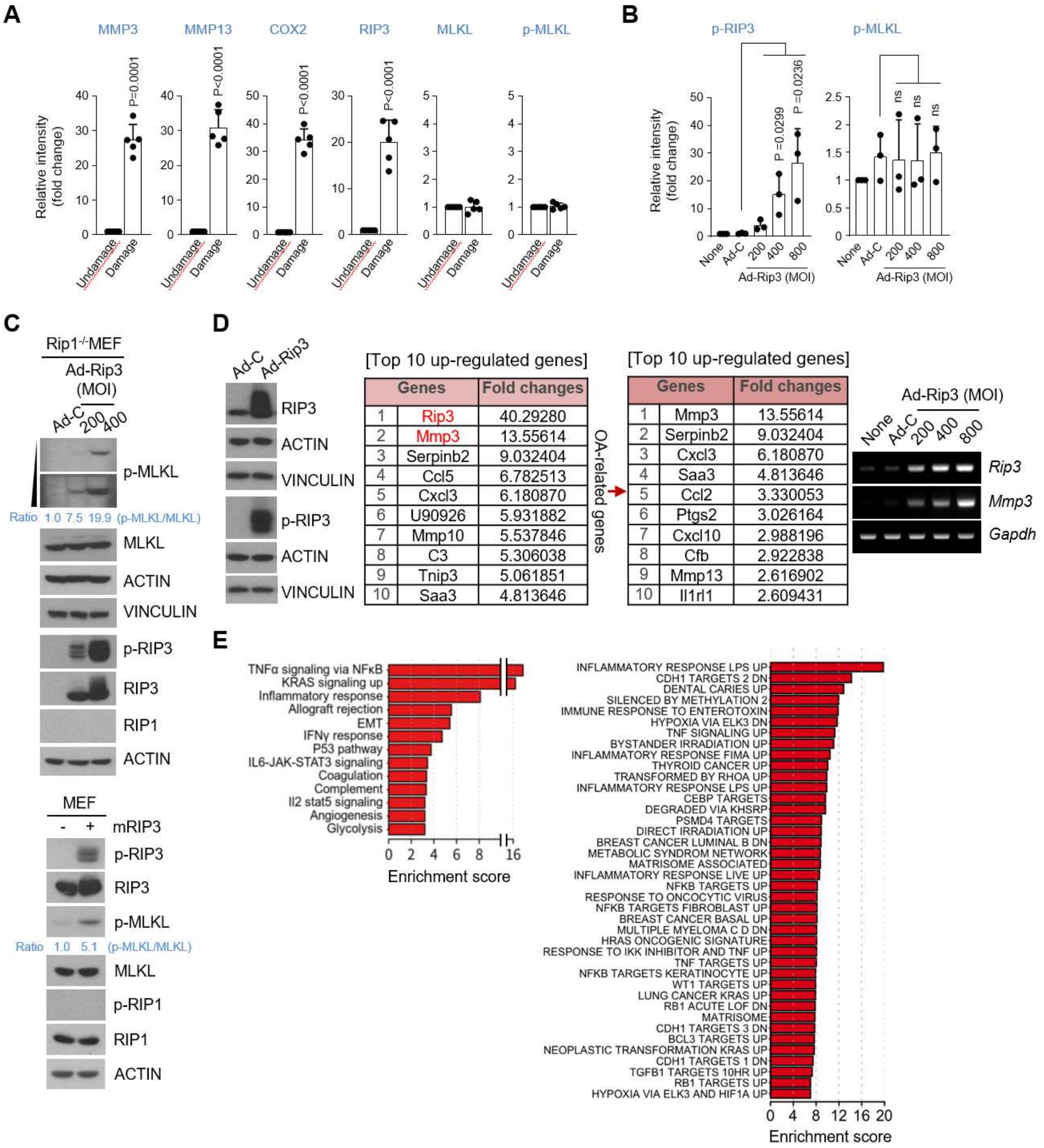

	Genes	Fold changes
1	Rip3	40.29280
2	Mmp3	13.55614
3	Serpinb2	9.032404
4	Ccl5	6.782513
5	Cxcl3	6.180870
6	U90926	5.931882
7	Mmp10	5.537846
8	C3	5.306038
9	Tnip3	5.061851
10	Saa3	4.813646

	Genes	Fold changes
1	Mmp3	13.55614
2	Serpinb2	9.032404
3	Cxcl3	6.180870
4	Saa3	4.813646
5	Ccl2	3.330053
6	Ptgs2	3.026164
7	Cxcl10	2.988196
8	Cfb	2.922838
9	Mmp13	2.616902
10	Il1rl1	2.609431

Figure S2. Elevated RIP3 expression correlated with OA pathogenesis-related gene expression patterns in chondrocytes. **(A)** Relative expression levels of the indicated proteins were determined from the immunohistochemistry of human undamaged or damaged cartilage in figure 1C (*n*=5). **(B)** Quantification of western blotting results in figure 1E (*n*=3). **(C)** Western blot analysis of necroptosis regulator in Ad-Rip3- and Ad-C-infected RIP1$^{-/-}$MEFs and Flag-RIP3 transfected MEFs (*n*=3). **(D)** Western blot (left) and gene expression (right) analysis of RIP3 and p-RIP3 in chondrocytes infected with Ad-C or Ad-Rip3. Differentially expressed genes (DEGs) between Ad-Rip3 and Ad-C-infected chondrocytes were selected using fold change (FC) > 3. Top 10 upregulated DEGs (Up-DEGs) and the top 10 OA-related Up-DEGs are listed (middle) by RIP3 overexpression. Functional annotation was significantly enriched in Up-DEGs. Hypergeometric tests were performed using hallmark gene annotation in MSigDB (http://software.broadinstitute.org/gsea/msigdb), yielding enrichment scores defined as $-\log_{10}$ (q-value). RT-PCR analysis of *Mmp3* in chondrocytes infected with Ad-C or Ad-Rip3 (right). **(E)** Significantly enriched functional terms in Up-DEGs. Functional annotation was obtained from the curated gene sets (C2) in MSigDB. Values are expressed as the mean ± SEM. Statistical analyses were performed using a two-tailed t-test.

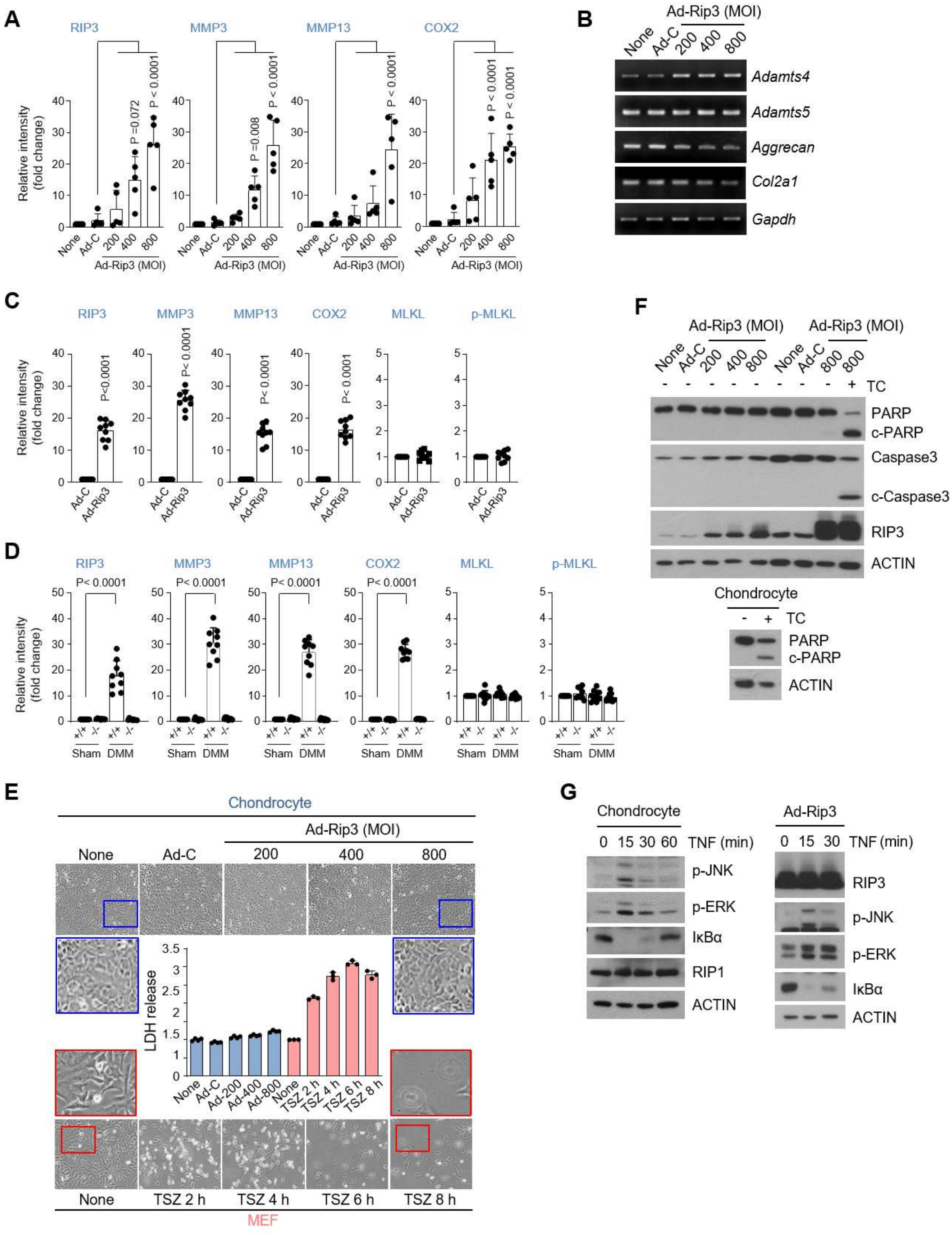

Figure S3. Elevated RIP3 expression does not induce chondrocyte cell death. (A and B) Relative expression levels of the western blotting results in figure 2B (**A**) and RT-PCR (**B**) analysis of the indicated protein or transcript levels in chondrocytes infected with Ad-C or Ad-Rip3 at the indicated MOI (*n*=5). (**C and D**) Relative expression levels of the indicated proteins were determined from immunohistochemistry in Ad-C or Ad-Rip3 injected cartilage in figure 2F (**C**) or cartilage of DMM-operated WT or Rip3 KO mice in figure 3B (**D**) (*n*=9). **(E)** Cell cytotoxicity was analyzed by LDH assay or phase-contrast microscopy in chondrocytes infected with Ad-C or Ad-Rip3 at the indicated MOI. Control MEFs were treated with TSZ for the indicated times and cell cytotoxicity analyzed by LDH assay or phase-contrast microscopy. **(F)** Analysis of apoptosis-related protein patterns in chondrocytes infected with Ad-C or Ad-Rip3 at the indicated MOI under apoptosis-induced conditions, TC (TNF 30 ng/mL + cycloheximide, CHX 1 mg/mL). Chondrocytes were also treated with TC without RIP3 overexpression, and cell lysates were analyzed by western blotting. (**G)** TNF-induced downstream signals in chondrocytes and RIP3 overexpressing chondrocytes. Cells were treated with TNF for indicated times, and cell lysates analyzed by western blotting. Values are presented as the means ± SEM and assessed using one-way ANOVA with Bonferroni's test (**A** and **D**) or a two-tailed t-test (**C**).

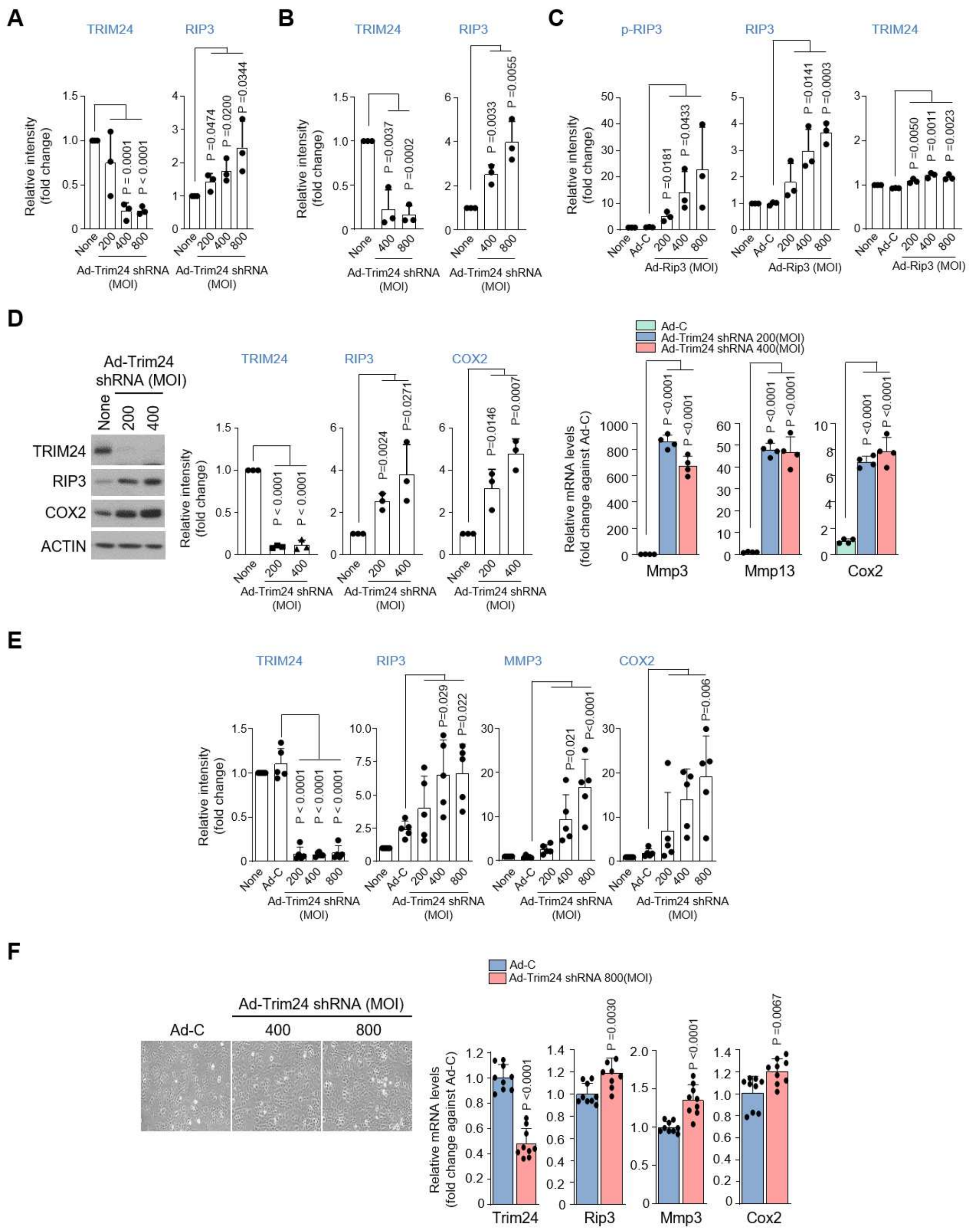

Figure S4. TRIM24 downregulation induced OA-related gene expression. (A-C) Quantification of western blotting results in figure 4B (**A**), figure 4C (**B**) and figure 4D (**C**) (*n*=3). **(D)** Western blot analysis of TRIM24, RIP3, and COX2 in MEFs infected with Ad-C or Ad-Trim24 shRNA at the indicated MOI (left) (*n*=3). Quantification of western blotting results presented as the means±SD based on three independent experiments (middle). qPCR analysis of *Mmp3, Mmp13*, and *Cox2* in MEFs infected with Ad-C or Ad-Trim24 shRNA (right) (*n*=4). **(E)** Relative expression levels of the indicated proteins were determined from western blot in chondrocytes infected with Ad-C or Ad-Trim24 shRNA at the indicated MOI in figure 5B (*n*=5). **(F)** Cell cytotoxicity was analyzed by phase-contrast microscopy (left) in chondrocytes infected with Ad-C or Ad-Trim24 shRNA at the indicated MOI. qPCR analysis of *Rip3, Mmp3, Cox2,* and *Trim24* in chondrocytes infected with Ad-C or Ad-Trim24 shRNA (right) (*n*=9). Values are expressed as the mean ± SEM. Statistical analyses were performed using a two-tailed *t*-test (**A**-**D**) or one-way ANOVA with Bonferroni's test (**E**).

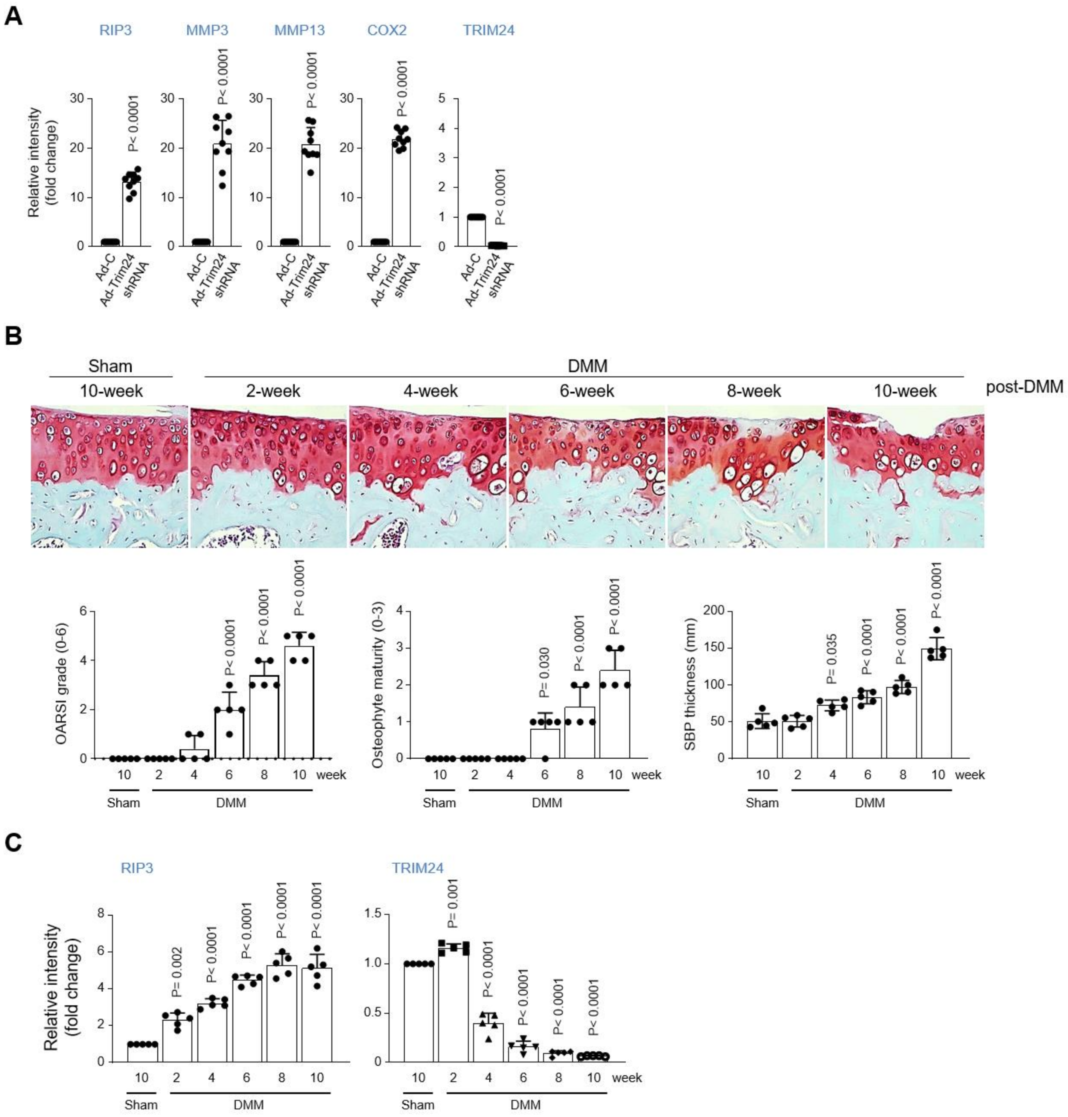
A
RIP3
MMP3
MMP13
COX2
TRIM24
Relative intensity (fold change)
P< 0.0001
Ad-C
Ad-Trim24 shRNA
B
Sham
DMM
10-week
2-week
4-week
6-week
8-week
10-week
post-DMM
OARSI grade (0-6)
Osteophyte maturity (0-3)
SBP thickness (mm)
P= 0.030
P= 0.035
week
C
RIP3
TRIM24
P= 0.002
P= 0.001

Figure S5. The expression of TRIM24 and RIP3 inversely correlated in a DMM-induced OA model. (**A**) Relative expression levels of the indicated proteins were determined from immunohistochemistry in chondrocytes infected with Ad-C or Ad-Trim24 shRNA at the indicated MOI in figure 5D (*n*=9). (**B** and **C**) WT mice were subjected to DMM surgery to induce cartilage destruction (*n*=5). Osteoarthritic manifestations were scored using the OARSI score and by assessing osteophyte and subchondral bone formation (**B**). Relative expression levels of the indicated proteins were determined from immunohistochemistry in DMM-operated cartilage in figure 5E (**C**). Values are expressed as the mean ± SEM. Data were analyzed using a two-tailed *t*-test (**A**) or one-way ANOVA with Bonferroni's test (**B** and **C**).

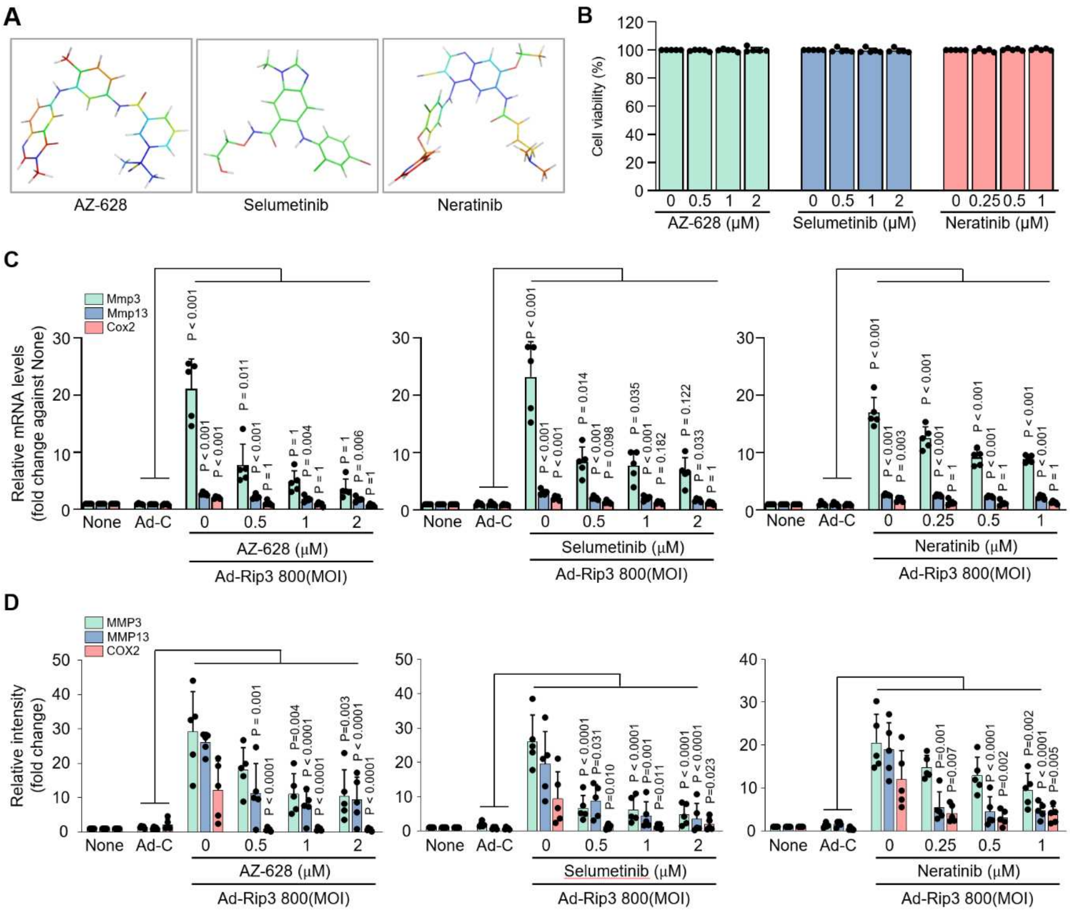

Figure S6. Selection of new inhibitors to regulate RIP3 kinase activity. (**A**) 3D structures of inhibitor molecules from PubChem. (**B**) Effects of AZ-628, selumetinib, and neratinib on chondrocyte viability detected by LDH assays (*n*=5). (**C** and **D**) Relative expression levels of the indicated proteins were determined from qPCR (**C**) and western blotting results in figure 6C (**D**) in chondrocytes infected with Ad-C or Ad-Rip3 in the presence or absence of AZ-628, selumetinib, and neratinib at the indicated dose (*n*=5). Values are expressed as the mean ± SEM. Data were analyzed using one-way ANOVA with Bonferroni's test.

A

Compound	PubChem CID	Molecular Formula	Binding Affinity, ΔG (kcal/mol)
Dabrafenib	44462760	$C_{23}H_{20}F_3N_5O_2S_2$	-8.6
Erk-inhibitor	135523966	$C_{22}H_{17}Cl_2FN_4O_2$	-8.4
AZ-628	11676786	$C_{27}H_{25}N_5O_2$	-8.4
Geldanamycin	5288382	$C_{29}H_{40}N_2O_9$	-7.9
Trametinib	11707110	$C_{26}H_{23}FIN_5O_4$	-7.8
Neratinib	9915743	$C_{30}H_{29}ClN_6O_3$	-7.6
HS-1371	134817449	$C_{24}H_{24}N_4O$	-7.6
PD98059	4713	$C_{16}H_{13}NO_3$	-7.1
PD0325901	9826528	$C_{16}H_{14}F_3IN_2O_4$	-7.0
GSK872	54674134	$C_{19}H_{17}N_3O_2S_2$	-6.6
AZD8330	16666708	$C_{16}H_{17}FIN_3O_4$	-6.4
AS605240	5289247	$C_{12}H_7N_3O_2S$	-6.3
PD184352	6918454	$C_{17}H_{14}ClF_2IN_2O_2$	-5.9
Selumetinib	10127622	$C_{17}H_{15}BrClFN_4O_3$	-5.7

B

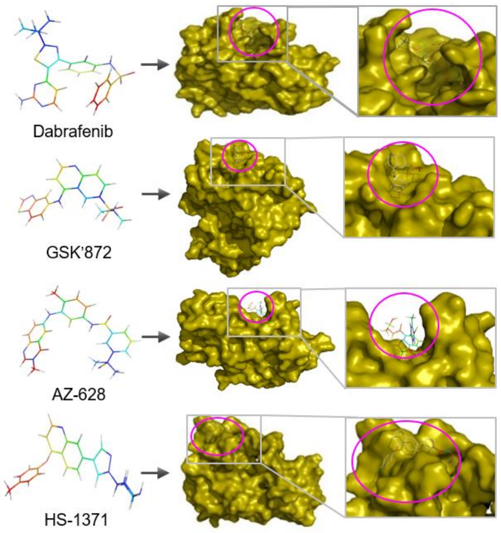

C

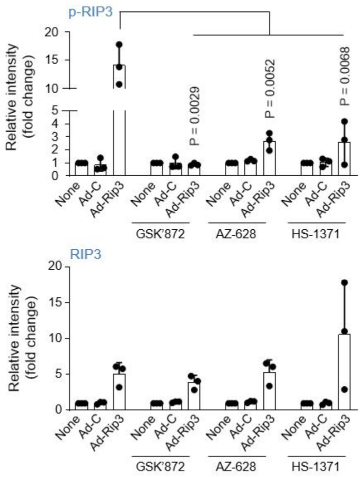

D

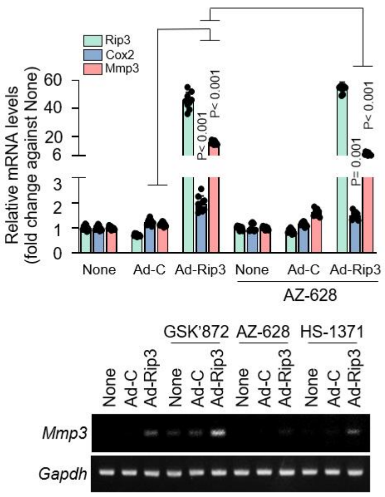

E

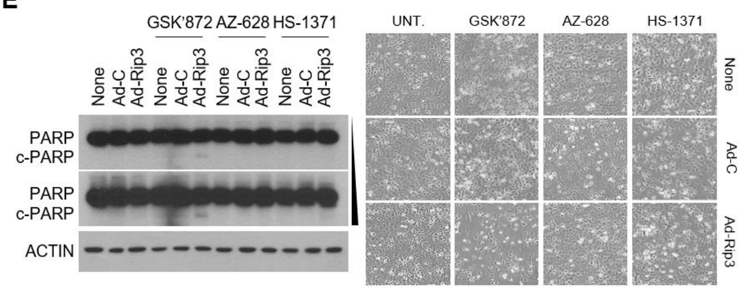

F

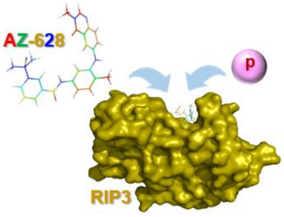

Figure S7. AZ-628 acts as a potent inhibitor of RIP3-mediated OA pathogenesis. (**A**) Binding affinity assay of 14 compounds with RIP3. (**B**) Computational docking model for RIP3 and small molecules. (**C**) Quantification of western blotting results in figure 6D (*n*=3). (**D**) qPCR analysis of *Rip3, Cox2,* and *Mmp3* expression in chondrocytes infected with Ad-C or Ad-Rip3 (n=9). (**E**) Cell cytotoxicity assessed by western blotting (left) and phase-contrast microscopy (right) in chondrocytes infected with Ad-C or Ad-Rip3. (**F**) Diagram of AZ-628 or phosphate binding to the RIP3 kinase domain. Values are expressed as the mean ± SEM. Statistical analyses were performed using a two-tailed *t*-test.

SUPPLEMENTARY REFERENCE

1. Jeon J, Kang LJ, Lee KM, et al. 3'-Sialyllactose protects against osteoarthritic development by facilitating cartilage homeostasis. *J Cell Mol Med* 2018;22(1):57-66. doi: 10.1111/jcmm.13292
2. Yang S, Kim J, Ryu JH, et al. Hypoxia-inducible factor-2alpha is a catabolic regulator of osteoarthritic cartilage destruction. *Nat Med* 2010;16(6):687-93. doi: 10.1038/nm.2153
3. Kim YS, Morgan MJ, Choksi S, et al. TNF-induced activation of the Nox1 NADPH oxidase and its role in the induction of necrotic cell death. *Mol Cell* 2007;26(5):675-87. doi: 10.1016/j.molcel.2007.04.021
4. Song EK, Jeon J, Jang DG, et al. ITGBL1 modulates integrin activity to promote cartilage formation and protect against arthritis. *Sci Transl Med* 2018;10(462) doi: 10.1126/scitranslmed.aam7486
5. Wang Y, Bryant SH, Cheng T, et al. PubChem BioAssay: 2017 update. *Nucleic Acids Res* 2017;45(D1):D955-D63. doi: 10.1093/nar/gkw1118
6. Trott O, Olson AJ. AutoDock Vina: improving the speed and accuracy of docking with a new scoring function, efficient optimization, and multithreading. *J Comput Chem* 2010;31(2):455-61. doi: 10.1002/jcc.21334 [published Online First: 2009/06/06]
7. Morris GM, Huey R, Lindstrom W, et al. AutoDock4 and AutoDockTools4: Automated docking with selective receptor flexibility. *J Comput Chem* 2009;30(16):2785-91.
8. Subramanian A, Tamayo P, Mootha VK, et al. Gene set enrichment analysis: A knowledge-based approach for interpreting genome-wide expression profiles. *Proceedings of the National Academy of Sciences of the United States of America* 2005;102(43):15545-50. doi: 10.1073/pnas.0506580102
9. Kim S, Han S, Kim Y, et al. Tankyrase inhibition preserves osteoarthritic cartilage by coordinating cartilage matrix anabolism via effects on SOX9 PARylation. *Nat Commun* 2019;10(1):4898. doi: 10.1038/s41467-019-12910-2
